# Health-related quality of life associated with trifluridine/tipiracil in heavily pretreated metastatic gastric cancer: results from TAGS

**DOI:** 10.1007/s10120-020-01053-9

**Published:** 2020-03-04

**Authors:** Josep Tabernero, Maria Alsina, Kohei Shitara, Toshihiko Doi, Mikhail Dvorkin, Wasat Mansoor, Hendrik-Tobias Arkenau, Aliaksandr Prokharau, Michele Ghidini, Catia Faustino, Vera Gorbunova, Edvard Zhavrid, Kazuhiro Nishikawa, Takayuki Ando, Şuayib Yalçın, Eric Van Cutsem, Javier Sabater, Donia Skanji, Catherine Leger, Nadia Amellal, David H. Ilson

**Affiliations:** 1 Vall d’Hebron University Hospital and Institute of Oncology (VHIO), UVic-UCC, IOB-Quiron, Barcelona, Spain; 2 National Cancer Center Hospital East, Chiba, Japan; 3 Omsk Regional Clinical Centre of Oncology, Omsk, Russia; 4 The Christie NHS Foundation Trust, Manchester, UK; 5 Sarah Cannon Research Institute, London, UK; 6 Minsk City Clinical Oncology Dispensary, Minsk, Belarus; 7 Azienda Ospedaliera di Cremona, Cremona, Italy; 8 Instituto Português de Oncologia do Porto Francisco Gentil, Porto, Portugal; 9 N.N. Blokhin Russian Cancer Research Center, Moscow, Russia; 10 Alexandrov National Cancer Centre of Belarus, Minsk, Belarus; 11 Osaka National Hospital, Osaka, Japan; 12 University of Toyama, Toyama, Japan; 13 Hacettepe University, Ankara, Turkey; 14 University Hospitals and KU Leuven, Leuven, Belgium; 15 Market Access Department, Servier, Suresnes, France; 16 Institut de Recherches Internationales Servier, Suresnes, France; 17 Memorial Sloan Kettering Cancer Center, New York, NY USA

**Keywords:** Gastric cancer, Health-related quality of life, Phase 3, Trifluridine/tipiracil

## Abstract

**Background:**

In TAGS, an international, double-blind, phase 3 trial, trifluridine/tipiracil significantly improved overall survival and progression-free survival compared with placebo in heavily pretreated metastatic gastric cancer patients. This paper reports pre-specified quality of life (QoL) outcomes for TAGS.

**Methods:**

Patients were randomized 2:1 to trifluridine/tipiracil (35 mg/m^2^ twice daily on days 1–5 and 8–12 of each 28-day cycle) plus best supportive care (BSC) or placebo plus BSC. QoL was evaluated at baseline and at each treatment cycle, using the EORTC QLQ-C30 and EORTC QLQ-STO22 questionnaires; results were considered valid for analysis only if ≥ 10% of patients completed the questionnaires. Key QoL outcomes were mean changes from baseline and time to deterioration in QoL. A post hoc analysis assessed the association between QoL and time to deterioration of Eastern Cooperative Oncology Group performance score (ECOG PS) to ≥ 2.

**Results:**

Of 507 randomized patients, 496 had baseline QoL data available. The analysis cut-off was 6 cycles for trifluridine/tipiracil and 3 cycles for placebo. In both treatment groups, there were no clinically significant deteriorations in the mean QLQ-C30 Global Health Status (GHS) score, or in most subscale scores. In a sensitivity analysis including death and disease progression as events, there was a trend towards trifluridine/tipiracil reducing the risk of deterioration of QoL scores compared with placebo. Deterioration in the GHS score was associated with deterioration in ECOG PS.

**Conclusion:**

QoL was maintained in TAGS, and there was a trend towards trifluridine/tipiracil reducing the risk of QoL deterioration compared with placebo.

*Trial registration* ClinicalTrials.gov number: NCT02500043

## Introduction

Worldwide, gastric cancer is the fifth most common cancer and the third leading cause of cancer-related death [1]. The majority of patients present with advanced or metastatic disease and the prognosis for these patients is relatively poor [2], with a 5-year overall survival (OS) of less than 30% [3]. After the failure of first- and second-line treatment, there are limited treatment options for patients with metastatic gastric cancer. Thus, there is a need for effective agents with manageable safety profiles.

Trifluridine/tipiracil is an oral combination of the thymidine-based nucleoside analogue, trifluridine, and the thymidine phosphorylase inhibitor tipiracil hydrochloride [4, 5]. In TAGS, the randomized, double-blind, phase 3 trial in patients with heavily pretreated metastatic gastric cancer, trifluridine/tipiracil significantly improved median OS compared with placebo (5.7 vs 3.6 months), with a 31% reduction in risk of death (HR: 0.69; 2-sided *P *= 0.0006) [6]. Trifluridine/tipiracil was also associated with significant improvements in progression-free survival (PFS; 2.0 vs 1.8 months; HR: 0.57; 2-sided *P *< 0.0001) and time to Eastern Cooperative Oncology Group performance score (ECOG PS) deterioration to ≥ 2 (31% reduction in risk, HR: 0.69; 2-sided *P *= 0.0005) compared with placebo, and demonstrated a predictable and manageable safety profile [6]. Based on the results of TAGS, trifluridine/tipiracil was approved in the USA, the EU, and Japan for third-line treatment of metastatic gastric or gastroesophageal junction (GEJ) adenocarcinoma in adult patients [7–9].

Disease symptoms and drug toxicity can have a negative impact on patients’ quality of life (QoL); therefore, in addition to OS, QoL is an important outcome to measure in trials in patients with cancer [10]. This is particularly true for patients with advanced cancer who may have a limited life expectancy, in which case any survival benefits must be weighed against treatment toxicity and impact on QoL [10]. Evaluation of QoL includes patient-reported physical, psychological and social dimensions, and best reflects how patients perceive their own state of health. In this paper, we report the effect of trifluridine/tipiracil versus placebo on patient-reported QoL, evaluated as a pre-specified endpoint in TAGS.

## Methods

### Study design

TAGS (ClinicalTrials.gov number: NCT02500043) was an international, randomized, double-blind, placebo-controlled, phase 3 trial in patients (aged ≥ 18 years) with pre-treated (≥ 2 regimens), histologically confirmed, non-resectable metastatic gastric adenocarcinoma, including adenocarcinoma of the gastroesophageal junction. Full study design details have been published previously [6]. Briefly, eligible patients were randomized 2:1 to receive either oral trifluridine/tipiracil 35 mg/m^2^ twice daily plus best supportive care (BSC) or placebo twice daily plus BSC on days 1–5 and 8–12 of each 28-day cycle. Previous regimens must have included a fluoropyrimidine, a platinum agent, and a taxane or irinotecan, or both. Patients whose tumors were HER2 positive must have received previous anti-HER2 therapy, if available. Randomization was stratified by region (Japan vs rest of World), ECOG PS (0 vs 1), and previous treatment with ramucirumab (yes vs no). Treatment continued until disease progression, unacceptable toxicity or patient withdrawal.

The study protocol was approved by the appropriate ethical review committees; the study was performed in accordance with the Declaration of Helsinki and Good Clinical Practice and all patients provided written, informed consent.

### QoL assessments

QoL was a pre-specified secondary endpoint of TAGS and was evaluated using two validated, reliable and sensitive questionnaires [11]: the European Organization for Research and Treatment of Cancer (EORTC) Quality of Life Questionnaire-Core 30 (QLQ-C30) and the EORTC Quality of Life Questionnaire-Gastric Cancer Module (QLQ-STO22). EORTC QLQ-C30 was developed to assess the QoL of a wide range of cancer patients and incorporates a Global Health Status (GHS) scale, five functional scales (physical, role, cognitive, emotional and social), three symptom scales (fatigue, pain and nausea or vomiting) and six single items assessing additional symptoms commonly reported by cancer patients (dyspnea, loss of appetite, insomnia, constipation, diarrhea) and perceived financial impact of the disease [12]. The gastric cancer-specific questions on the EORTC QLQ-STO22 include four single-item subscales (dry mouth, body image, hair loss and problems with taste) and five multi-item subscales (dysphagia, dietary restriction, pain, upper gastroesophageal symptoms and emotional problems) [13]. This 22-item instrument was used alongside the QLQ-C30, resulting in a total of 52 items. A high score for a functional scale or global health item represents a better QoL (high level of functioning) whereas a high symptom score indicates a poorer QoL, i.e. a high level of symptoms.

QoL data were collected 1–7 days before randomization (baseline), prior to treatment administration on day 1 of each cycle (from cycle 2 onwards), and at the 30-day safety follow-up visit (if not performed in the past 4 weeks). The key pre-specified QoL outcomes were mean changes from baseline and time to deterioration in QoL. A post hoc analysis of the association between QoL and time to deterioration of ECOG PS to ≥ 2 was also performed.

### Statistical analyses

Analyses were conducted in all randomized patients who completed at least one baseline and post-baseline EORTC QLQ-C30 or QLQ-STO22 questionnaire. The compliance rate was calculated using the proportion of patients having completed a QoL questionnaire and the proportion of patients remaining in each cycle.

Mean changes in scores from baseline to each cycle were determined. Descriptive statistics for both multi-item scales and single-item measures were provided for each assessed time point. Statistics included number of patients, number of non-missing/missing scales, means with standard deviation (SD) and medians with range. For each time cycle, results were considered valid for analysis only if ≥ 10% of the intention to treat (ITT) patient population (all randomized patients) completed the questionnaires (calculated separately for each treatment arm). For both questionnaires, a mean change from baseline of at least 10 points was considered to be clinically relevant, while a change of at least 5 points was considered “a little change” [14].

For the median time to deterioration in QoL, 95% confidence intervals (CI) were calculated; hazard ratios (HR) were calculated for between-group differences. Time to first deterioration in QoL was evaluated for each arm using Kaplan–Meier estimates and compared using the log-rank test. The main analysis of time to deterioration in QoL was defined as time to first deterioration by 5 points or more from baseline. Patients with no confirmed deterioration from baseline were censored at the time of their last observation. For this analysis, a Cox’s proportional hazard model was used to adjust for baseline EORTC QLQ-C30 and QLQ-STO22 scores, country and primary tumor type. Additionally, two sensitivity analyses were conducted. In the first, time to deterioration was defined as the first deterioration of ≥ 10 points from baseline, and death was considered to be a deterioration event. The second sensitivity analysis also considered a ≥ 10-point deterioration and included death or progressive disease (PD) without previous deterioration in QoL as deteriorative events. These analyses used Cox proportional hazards models adjusting for the randomization stratification factors (region, ECOG PS at baseline, prior treatment with ramucirumab).

A post hoc exploratory analysis investigated the association between time to deterioration of ECOG PS to ≥ 2 and changes in QoL throughout the study. To account for the longitudinally collected QoL scores, Cox proportional hazard models with time-dependent covariates were fitted to time-to-event data.

## Results

### Baseline characteristics and questionnaire compliance

The study was conducted in 110 academic hospitals in 17 countries. Between 24 February 2016 and 5 January 2018, 507 patients were enrolled and randomly assigned to receive trifluridine/tipiracil (*n* = 337) or placebo (*n* = 170). Baseline patient demographics and disease characteristics of the total patient population have been reported previously and were generally well balanced between the two groups [6]. Baseline QoL data was available for 332 (98.5%) patients in the trifluridine/tipiracil group and 164 (96.5%) patients in the placebo group.

Baseline compliance was similar between the two treatment groups; the number of patients completing the questionnaires decreased with each cycle, as the number of patients discontinuing treatment increased. The analysis cut-off point (i.e. including results only for time points at which ≥ 10% of the ITT population completed the questionnaires) was 6 cycles of treatment for trifluridine/tipiracil and 3 cycles for placebo (Table 1).

**Table 1:** Quality of Life questionnaire compliance rates for treatment cycles 1–6

	Rate of patients with QLQ-C30 questionnaire completed out of number of patients in the cycle		Rate of patients with QLQ-STO22 questionnaire completed out of number of patients in the cycle	
	Trifluridine/tipiracil (*n* = 337)	Placebo (*n* = 170)	Trifluridine/tipiracil (*n* = 337)	Placebo (*n* = 170)
Baseline	98.5	96.5	98.5	96.4
Cycle 1	84.2	76.8	84.1	76.8
Cycle 2	68.1	47.2	68.1	46.8
Cycle 3	85.5	69.7	85.3	69.7
Cycle 4	69.8	83.3	69.8	83.8
Cycle 5	86.2	66.7	86.2	66.7
Cycle 6	67.9	90.0	67.9	90.0

EORTC, European Organization for Research and Treatment of Cancer; QLQ-C30, Quality of Life Questionnaire-Core 30; QLQ-STO22, Quality of Life Questionnaire-Gastric Cancer Module

The mean baseline GHS, functioning and symptom scores were well balanced between the two treatment groups, with no differences of > 10 points on either questionnaire (Table 2). For both trifluridine/tipiracil and placebo, the mean baseline QLQ-C30 GHS score was 58.4 (SD for trifluridine/tipiracil, 20.2; for placebo, 19.7). On the QLQ-C30 questionnaire, the most common symptoms were fatigue, pain and appetite loss, and on the QLQ-STO22, they were anxiety, body image and hair loss (Table 2).

**Table 2:** Baseline quality of life scores

	Baseline score, mean (SD)	
	Trifluridine/tipiracil (*n* = 327–332^a^)	Placebo (*n* = 162–164^a^)
QLQ-C30		
GHS	58.4 (20.23)	58.4 (19.72)
Functioning scores^b^		
Physical	76.2 (18.92)	77.6 (18.11)
Role	75.9 (26.61)	77.1 (24.71)
Emotional	77.5 (21.31)	79.9 (18.30)
Cognitive	85.1 (18.75)	86.7 (17.87)
Social	79.4 (23.98)	79.6 (22.85)
Symptom scores^c^		
Fatigue	35.9 (21.08)	35.9 (22.86)
Nausea and vomiting	11.6 (18.93)	10.9 (19.01)
Pain	27.0 (25.65)	28.8 (26.32)
Dyspnea	15.9 (23.27)	17.3 (22.90)
Insomnia	24.4 (27.94)	22.3 (27.98)
Appetite loss	27.8 (29.24)	26.6 (29.58)
Constipation	14.6 (22.73)	18.4 (26.23)
Diarrhea	13.2 (22.56)	9.0 (18.91)
Financial difficulties	17.7 (25.34)	17.6 (26.53)
QLQ-STO22^c^		
Dysphagia	11.4 (17.46)	11.1 (18.45)
Dietary restrictions	21.0 (19.51)	21.3 (21.20)
Pain	23.3 (20.79)	22.9 (20.75)
Upper gastroesophageal	14.4 (18.04)	14.9 (17.82)
Anxiety	41.7 (24.20)	43.6 (25.94)
Dry mouth	21.9 (25.44)	21.7 (25.54)
Body image	26.4 (29.65)	30.7 (30.76)
Hair loss	26.8 (31.75)	23.3 (27.19)
Taste problems	19.1 (25.75)	17.4 (24.94)

GHS, Global Health Status; QLQ-C30, Quality of Life Questionnaire-Core 30; QLQ-STO22, Quality of Life Questionnaire-Gastric Cancer Module; SD, standard deviation

^a^Patient numbers varied slightly, depending on questionnaire/domain

^b^A high function score represents a better QoL

^c^A high symptom score represents a poorer QoL

### Change in QoL from baseline

**Fig. 1:**
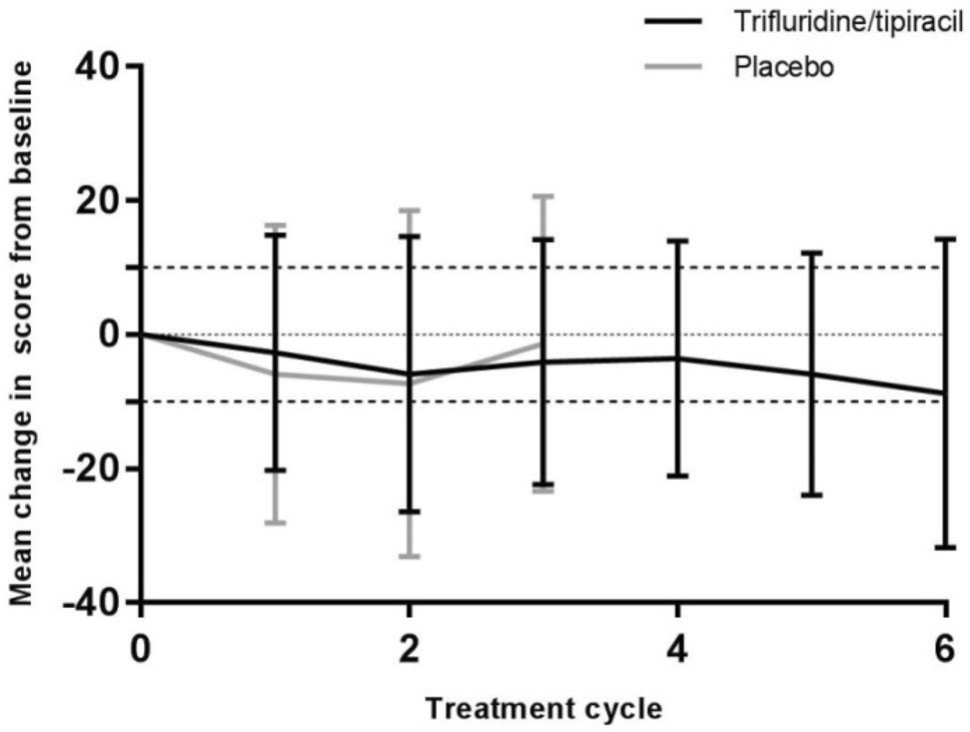
Change from baseline across treatment cycles* in the EORTC QLQ-C30 GHS score EORTC, European Organization for Research and Treatment of Cancer; GHS, global health status; QLQ-C30, Quality of Life Questionnaire-Core 30. *Results were considered valid for analysis only if ≥ 10% of the original patient population completed the questionnaires; this corresponded to 3 cycles of treatment with placebo and 6 cycles for trifluridine/tipiracil. A high score represents a high quality of life. A mean change from baseline of ≥ 10 points is considered clinically relevant

**Fig. 2:**
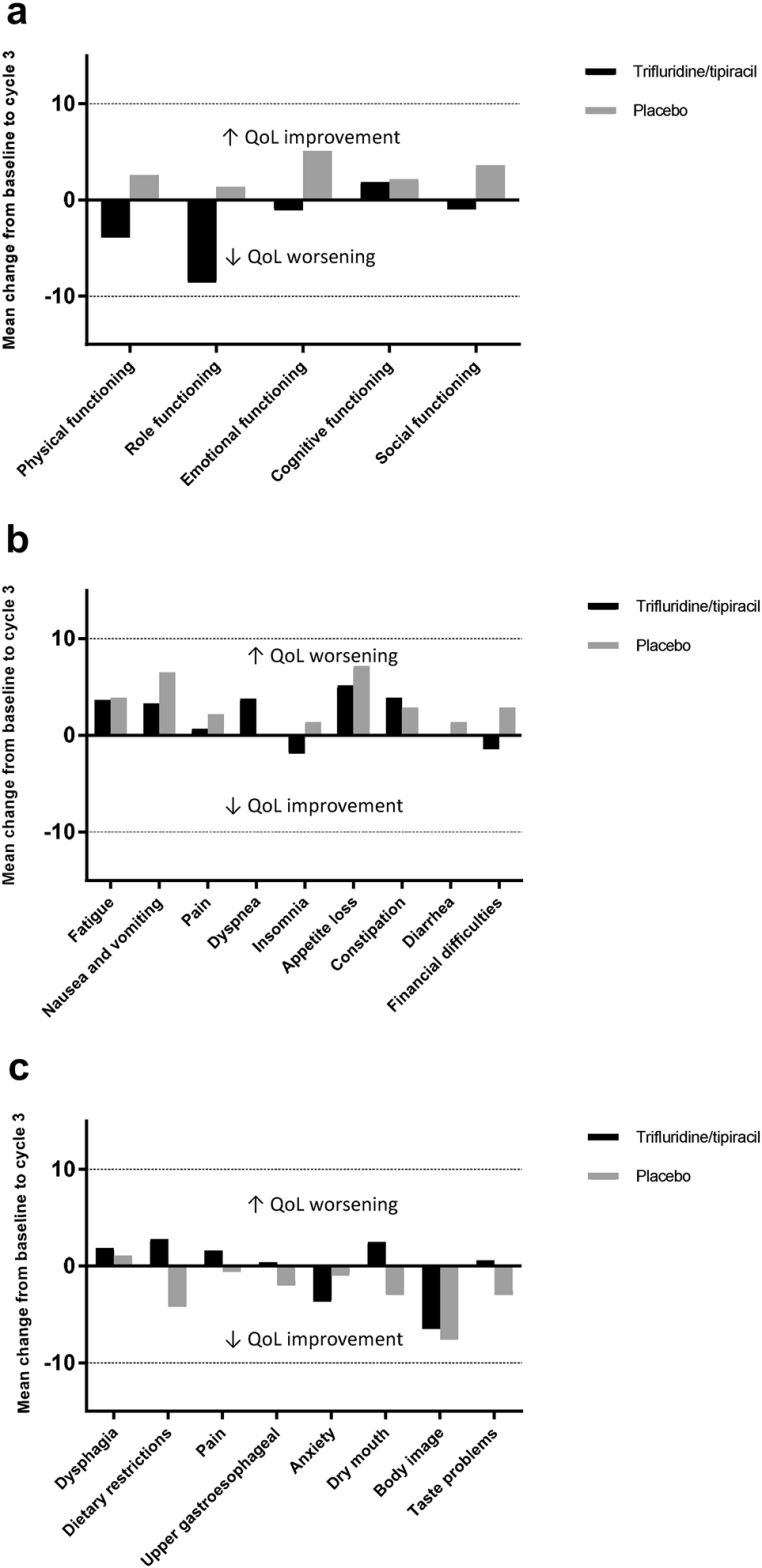
Change from baseline to treatment cycle 3 in the EORTC (**a**) QLQ-C30 function scores (**b**) QLQ-C30 symptom scores and (**c**) QLQ-STO22 subscores. EORTC, European Organization for Research and Treatment of Cancer; GHS, Global Health Status; QLQ-C30, Quality of Life Questionnaire-Core 30; QLQ-STO22, Quality of Life Questionnaire-Gastric Cancer Module

Although there was slight deterioration from baseline during treatment in both groups, there were no clinically relevant changes (≥ 10 points) in the mean QLQ-C30 GHS scores (Fig. 1) or in most of the subscale scores at any time point for which there were sufficient data (Fig. 2). With trifluridine/tipiracil, there were no clinically relevant deteriorations in any of the subscale scores from baseline until end of cycle 6, with the exception of deteriorations in the mean score of role functioning from baseline to the end of cycles 4 and 6 (−10.2 ± 24.2 and −13.4 ± 30.0, respectively). Clinically relevant deteriorations were seen in the placebo arm in the role functioning score from baseline to end of cycles 1 and 2, fatigue and pain scores from baseline to end of cycle 2, and appetite loss from baseline to end of cycle 1.

No clinically relevant differences (≥ 10 points) between treatment groups over time were observed, with the following exceptions: the QLQ-C30 pain score at end of cycle 2 (change from baseline was 11.3 points higher for trifluridine/tipiracil than placebo) and the role functioning score at end of cycle 3 (the placebo score improved from baseline by 1.4 points and the trifluridine/tipiracil score deteriorated from baseline by 8.6 points).

### Time to deterioration in QoL scores

In the main analysis, the median time to deterioration by ≥ 5 points in the QLQ-C30 GHS score was 2.6 months (95% CI 2.3, 3.3) for trifluridine/tipiracil and 2.3 months (95% CI 1.4–not estimable) for placebo (HR 1.27; 95% CI 0.85–1.87).

**Fig. 3:**
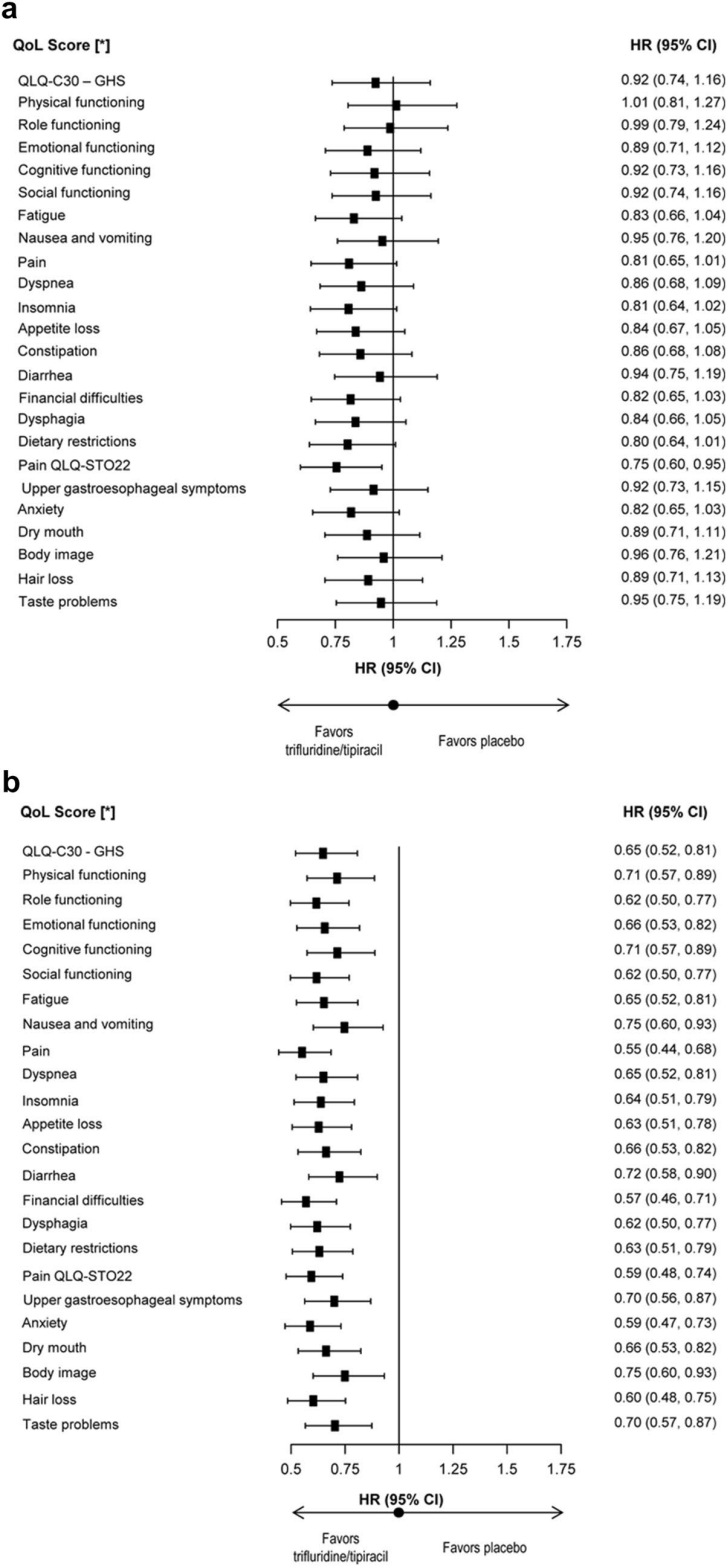
Time to deterioration in EORTC QoL scores by ≥ 10 points: sensitivity analyses including (**a**) death as an event and (**b**) disease progression and death as events. CI, confidence interval; EORTC, European Organization for Research and Treatment of Cancer; GHS, global health status; HR, hazard ratio; ITT, intention to treat; QLQ-C30, Quality of Life Questionnaire-Core 30; QLQ-STO22, Quality of Life Questionnaire-Gastric Cancer Module; QoL, quality of life. *Stratification factors were baseline Eastern Cooperative Oncology Group performance status (ECOG PS 0 vs 1) status, prior treatment with ramucirumab and region (Japan vs rest of World)

In the sensitivity analysis including death as an event, the risk of deterioration (by ≥ 10 points) in the QLQ-C30 GHS score was numerically lower with trifluridine/tipiracil than placebo (HR 0.92, 95% CI 0.74–1.16); similar findings were observed for all QLQ-C30 and QLQ-STO22 scores, with the exception of the physical functioning score (Fig. 3a). In this analysis, the median time to deterioration (by ≥ 10 points) in the QLQ-C30 GHS was 3.19 months (95% CI 2.80–3.82) for trifluridine/tipiracil and 2.27 months (95% CI 2.07–3.36) for placebo.

In the second sensitivity analysis which included death or PD as an event, compared with placebo, trifluridine/tipiracil reduced the risk of deterioration (by ≥ 10 points) for all QLQ-C30 and QLQ-STO22 scores (HRs ranged from 0.55 to 0.75; Fig. 3b). For the QLQ-C30 GHS score, the median time to deterioration in this analysis was 2.11 months (95% CI 2.07–2.27) for trifluridine/tipiracil versus 1.88 months (95% CI 1.84–1.94) for placebo (HR 0.65, 95% CI 0.52–0.81; Fig. 3b).

### Association between QoL and time to ECOG deterioration

**Fig. 4:**
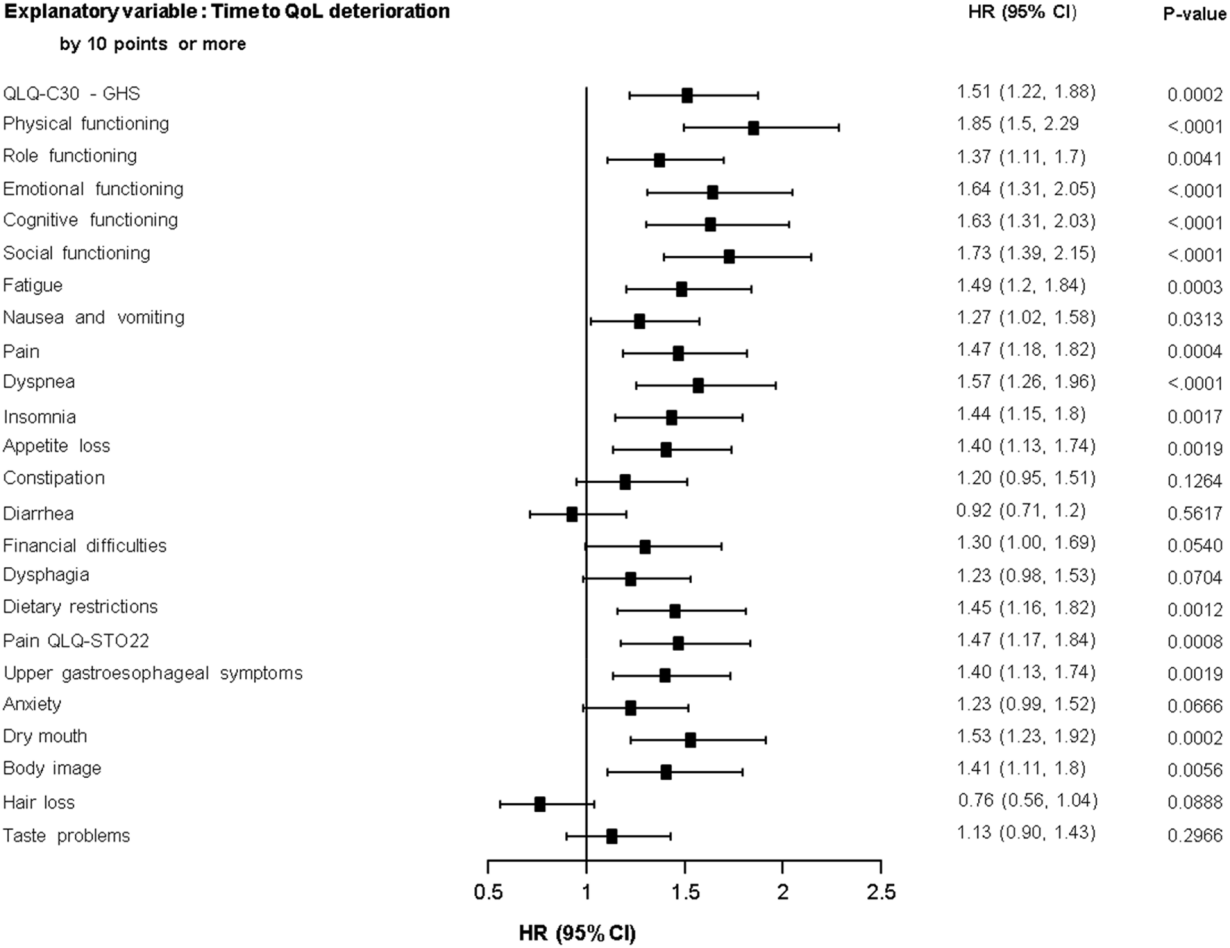
Association between time to deterioration of ECOG PS to ≥ 2 and time to deterioration of QoL score by ≥ 10 points. CI, confidence interval; EORTC, European Organization for Research and Treatment of Cancer; GHS, global health status; HR, hazard ratio; ITT, intention to treat; QLQ-C30, Quality of Life Questionnaire-Core 30; QLQ-STO22, Quality of Life Questionnaire-Gastric Cancer Module; QoL, quality of life

Deteriorations in QoL scores of ≥ 10 points were associated with a significantly increased risk of deterioration in ECOG PS to ≥ 2 for the QLQ-C30 GHS score, all QLQ-C30 functional scale scores, the QLQ-C30 fatigue, nausea and vomiting, pain, dyspnea, insomnia and appetite loss scores, and the QLQ-STO22 dietary restrictions, pain, upper gastroesophageal, dry mouth and body image scores (HRs ranged from 1.27–1.85; Fig. 4). A reduction in the QLQ-C30 GHS score of 10 points increased the risk of ECOG PS deterioration by 51% (HR 1.5, 95% CI 1.2–1.9).

## Discussion

In patients with advanced refractory gastric cancer, for whom QoL has most likely already been diminished by disease progression and previous treatments, maintaining QoL is an important treatment goal. Indeed, QoL is included alongside OS in the European Society for Medical Oncology Magnitude of Clinical Benefit Scale (ESMO-MCBS), a proposed tool for measuring the potential clinical benefit of new anti-cancer therapies [15].

In this analysis of QoL data for TAGS, QoL was maintained from baseline for patients with pre-treated (≥ 2 regimens), histologically confirmed, non-resectable metastatic gastric adenocarcinoma, including adenocarcinoma of the gastroesophageal junction, who were receiving trifluridine/tipiracil or placebo. As assessed by two widely used, validated, reliable and sensitive questionnaires (EORTC QLQ-C30 and QLQ-STO22), QoL for patients receiving trifluridine/tipiracil remained stable for all functional and symptom scales across treatment cycles 1–3, and all scores except role functioning across cycles 4–6. There were no clinically relevant changes in the QLQ-C30 GHS score during treatment, nor were there any clinically relevant differences between treatment groups in this score over time. When death was considered as an event, there was a trend in favor of treatment with trifluridine/tipiracil. Although statistical significance was not achieved, patients treated with trifluridine/tipiracil achieved numerically lower QoL changes than patients in the placebo group. Significant differences were observed in the sensitivity analysis including death or PD without previous deterioration in QoL as events. When both death and PD were considered as events, compared with placebo, patients treated with trifluridine/tipiracil achieved numerically lower changes in each QoL scores. By making the reasonable assumption that death and PD are associated with a decrease in QoL, time to deterioration (TTD) including deaths and PD as events might better capture the change in QoL and takes the missing data into account in a specific manner. The analyses of TTD in QoL scores showed comparable findings with an overall trend towards improvement with trifluridine/tipiracil compared with placebo.

These QoL results can be added to the previously published efficacy and safety results of TAGS, in which, compared with placebo, trifluridine/tipiracil significantly improved OS, PFS and the proportion of patients achieving disease control and had a predictable and manageable safety profile [6]. The most common (≥ 20%) adverse events experienced by patients receiving trifluridine/tipiracil were nausea, anemia, decreased appetite, vomiting, diarrhea and fatigue [6]. In the current analysis, fatigue and appetite loss were among the most severe of the QoL symptoms reported at baseline. However, there were no clinically relevant deteriorations in scores for these symptoms over the treatment period.

There are very few phase 3 trials with QoL data in similar patient populations as was studied in TAGS (i.e. patients with advanced gastric cancer who have received at least two previous lines of treatment). Key trials in similar patients include ATTRACTION-2 (third- or greater-line nivolumab vs placebo [16]), and JAVELIN Gastric 300 (third-line avelumab vs chemotherapy [17]); however, none of these trials reported a QoL analysis.

Limited QoL results were reported for a phase 3, randomized, double-blind study which compared third-line apatinib with placebo in Chinese patients (*n* = 267) with advanced gastric or gastroesophageal cancer [18]. Using the EORTC QLQ-C30 only, no significant between-group differences were observed at any measured time point for any of the QoL scores [18]. No data were given for the change in QoL from baseline, making it difficult to compare the QoL results with those from the current study. Based on the positive efficacy and acceptable safety results from this trial, apatinib was approved in China for patients with advanced gastric or gastroesophageal cancer who have progressed or relapsed after chemotherapy [19]. QoL endpoints were also assessed in the multinational ANGEL study, in which third-line apatinib did not significantly improve OS compared with placebo [20, 21]; however, QoL results from this trial are not yet available.

A QoL sub-study of the phase 2 INTEGRATE study used the QLQ-C30, QLQ-STO22 and EuroQol-5D (EQ-5D) questionnaires as well as the patient disease and treatment (PTDATA) form to measure QoL in patients (*n* = 142) with advanced gastric cancer receiving second- or third-line regorafenib or placebo [22]. To interpret results in a clinically meaningful way, both INTEGRATE and the current TAGS analyzed only results for which at least 10% of the original patient population completed QoL questionnaires. Using this approach, the TAGS data are robust enough to allow analysis for 3 treatment cycles (≈ 12 weeks) for placebo and 6 cycles (≈ 24 weeks) for trifluridine/tipiracil. Similarly, in INTEGRATE, the 10% cut-off point corresponded to week 8 for placebo and week 16 for regorafenib [22]. In both INTEGRATE and the current TAGS, baseline QLQ symptom scores were highest for anxiety, fatigue, body image, and appetite loss [22]. At week 4 in INTEGRATE, the mean diarrhea score was significantly higher for regorafenib than placebo; however, there were no other between-group differences in QoL scores at week 4 or 8 [22]. Although the QoL scores tended to worsen for both groups from baseline to week 8 in INTEGRATE, the rate of deterioration-free survival was significantly longer for regorafenib than placebo, leading the authors to conclude that regorafenib did not have an excessively negative effect on QoL [22]. When analyzing the prognostic value of baseline QoL scores for OS, adjusting for treatment allocation, OS was longer in patients with lower baseline scores for several symptoms (general pain, abdominal pain, appetite loss, constipation and eating restrictions), and higher baseline scores for physical functioning, role functioning and EQ-5D utility [22].

Although physicians intuitively associate disease progression with a deterioration in QoL, until recently this association had not been studied specifically for patients with gastric cancer [23]. To address this knowledge gap, a pooled analysis of QLQ-C30 data was conducted for two global phase 3 trials (RAINBOW and REGARD) in patients with gastric cancer receiving second-line therapy [23]. In this analysis, disease progression and deterioration in ECOG PS scores resulted in worse QoL scores [23]. Furthermore, changes from baseline to 6 weeks in global and functional scale QoL scores, as well as those in fatigue, pain and appetite loss scores, were predictive of changes in tumor status. As would be expected, a small change in the physical functioning score predicted an ECOG PS change [23]. The authors noted that these results emphasize the importance of disease control for the maintenance of QoL and suggested that QoL may be an additional useful tool for assessing tumor status in patients with non-measurable disease [23]. In TAGS, disease control was achieved by significantly more patients receiving trifluridine/tipiracil than placebo (44% vs 14.5%, *p* < 0.0001) [6]. In the current analysis, clinically relevant deteriorations in the QLQ-C30 GHS score and in the majority of other QoL scores were significantly associated with an increased risk of deterioration in ECOG PS to ≥ 2.

Our study is not without limitations. The reduction in questionnaire completion rates over time meant it was not possible to assess the long-term effects of trifluridine/tipiracil treatment on QoL. Although the baseline questionnaire compliance rate was high, by treatment cycle 3 it was ≈ 85% for trifluridine/tipiracil and 70% for placebo; rates by cycle 6 had further decreased to 68% and 90%, respectively. This limitation is common in cancer QoL studies and can be explained by patients discontinuing treatment. In the above-mentioned trial of apatinib, questionnaire compliance rates at baseline and at the end of treatment cycles 2 and 3 were 100%, 60.8%, and 34.7% for apatinib recipients and 100%, 47.3% and 7.7%, respectively, for placebo recipients [18]. In the INTEGRATE sub-study, there were no post-baseline QoL data available for 29% of regorafenib recipients and 41% of placebo recipients [22].

## Conclusions

In this analysis of data from TAGS, QoL was maintained in patients with heavily pretreated, metastatic gastric cancer who received treatment with trifluridine/tipiracil, and there was a trend towards trifluridine/tipiracil reducing the risk of QoL deterioration compared with placebo. Together with previously published results showing that trifluridine/tipiracil has a manageable safety profile and gives prolonged OS compared with placebo, these data support trifluridine/tipiracil as a new treatment option in this difficult-to-treat patient population.
